# A case report of *Pseudomonas citronellolis* and *Escherichia coli* isolated from acute suppurative appendicitis: reveals the potential intestinal colonization and pathogenicity of *Pseudomonas citronellolis*

**DOI:** 10.3389/fcimb.2024.1280188

**Published:** 2024-02-16

**Authors:** Yugui Lin, Yanfen Li, Chunxiu Lin

**Affiliations:** ^1^Microbiology Laboratory, Zhongshan Bo’ai Hospital, Zhongshan, China; ^2^State Key Laboratory of Food Science and Technology, Jiangnan University, Wuxi, China

**Keywords:** *Pseudomonas citronellolis*, acute suppurative appendicitis, infection, intestinal colonization, pathogenicity

## Abstract

Human infections caused by *Pseudomonas citronellolis*, an environmental bacterium, are infrequent, with only two cases related to uncommon urinary tract infections and bacteremia reported in recent years. All these cases typically occurred in elderly patients with compromised or decreased immune function. Simultaneously, the epithelial barrier disruption induced by invasive biopsy procedures or gastrointestinal disorders such as gastroenteritis provided a pathway for *Pseudomonas citronellolis* to infiltrate the organism. In this study, we present the first report of a case where *Pseudomonas citronellolis* and *Escherichia coli* were isolated from the inflamed appendix of a patient without underlying conditions. Compared to the *Escherichia coli*, *Pseudomonas citronellolis* has never been isolated in patients with appendicitis. We identified the species using MALDI-TOF MS and genetic sequencing. Based on our findings, we highlight the perspective that *Pseudomonas citronellolis* can colonize the intestines of healthy individuals and may trigger infections like appendicitis.

## 1 Introduction

The *Pseudomonas* genus, which includes species such as *Pseudomonas aeruginosa*, *Pseudomonas putida*, *Pseudomonas fluorescens*, *Pseudomonas stutzeri*, *Pseudomonas mendocina*, *Pseudomonas alcaligenes*, and *Pseudomonas pseudoalcaligenes*, has frequently been isolated from human hosts and is often regarded as opportunistic pathogens (Peix et al., 2018). Nonetheless, infections attributed to *Pseudomonas citronellolis* (*P. citronellolis*), an environmental bacterium, are infrequent, with merely two reported cases in recent years (Williams, 2019; Hwang et al., 2023). Additionally, infections caused by it are typically opportunistic and linked to risk factors such as aging, compromised immunity, and skin or visceral mucosal damage, as documented in previous literature (Williams, 2019; Hwang et al., 2023). This study presents the first report of a case involving acute suppurative appendicitis due to co-infection with *P. citronellolis* and *Escherichia coli* (*E. coli*), with a total bacterial count exceeding 1×10^^6^ colony-forming units (CFU)/mL (≥+++) using a semi-quantitative approach (He, 2006; Klug et al., 2011). *E. coli* is commonly found in non-sterile appendicitis as an opportunistic pathogen (Plattner et al., 2021), however, it is crucial not to ignore the threat posed by *P. citronellolis* in this case. Given the bacterial load of *P. citronellolis*, which was comparable to that of *E. coli* in the sample, and the reported infections associated with *P. citronellolis* (Williams, 2019; Hwang et al., 2023), it is necessary to consider its potential pathogenicity. Notably, in this case, the patient lacked potential risk factors such as immune insufficiency, aging, or invasive surgical procedures. Fortunately, the patient achieved full recovery following an appendectomy and antibiotic therapy. This study will advance our comprehension of the potential pathogenicity of *P. citronellolis*.

## 2 Case presentation

**Figure 1:**
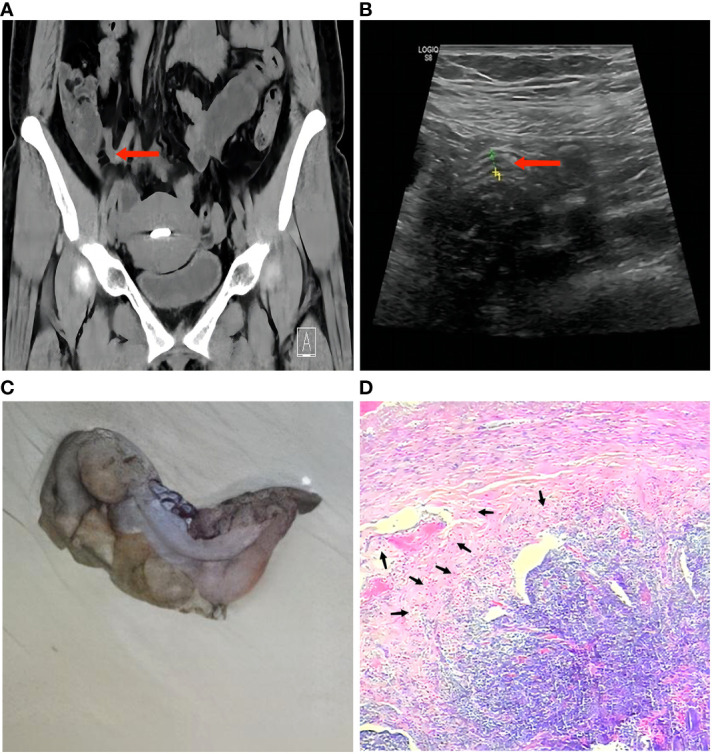
Imaging and pathological examinations of the patient. **(A)** An abdominal CT examination showed that the appendix was swollen and thickened, accompanied by blurred fat space and a little exudation. **(B)** An abdominal color Doppler examination showed that the appendix was swollen and thickened. **(C)** The swollen appendix was removed, with a length of 10 centimeters and a body diameter of 0.6 centimeter. **(D)** Histopathological examination revealed a significant presence of inflammatory exudate and infiltration of inflammatory cells within the appendiceal cavity, accompanied by abundant blood clots and extensive Gram-negative bacilli colonization. The red arrow indicates the swollen appendix, and the black arrow indicates the distribution of Gram-negative bacilli.

A 38-year-old female was admitted to our hospital for evaluation of severe pain in the lower abdomen, which had persisted for half a day but was not accompanied by vomiting or diarrhea. The patient was conscious and in good nutritional status. This female had a normal menstrual cycle, no history of chronic diseases such as diabetes or hypertension, no known allergies, and no recorded infectious diseases like tuberculosis, typhoid fever, hepatitis, etc. A physical examination revealed a body temperature of 36.5°C, blood pressure of 110/86 mmHg, pulse rate of 88 beats per minute, and respiratory rate of 20 breaths per minute. The patient’s skin, mucous membranes, and sclera showed no signs of jaundice or swelling of superficial lymph nodes. Abdominal examination indicated tenderness and rebound pain, particularly at McBurney’s point. Physical examinations of the heart, lungs, liver, spleen, and nervous system revealed no abnormalities. Laboratory tests showed an elevated white blood cell count of 11.12×10^^9^/L, with neutrophils accounting for 74.9%, platelets at 125×10^^9^/L, and a hemoglobin level of 125 g/L. Biochemical analysis displayed increased levels of C-reactive protein at 61.04 mg/L and procalcitonin at 137 ng/mL. The serum electrolytes, liver and kidney function, immunoglobulins, and complement were all within the normal ranges. Urine and fecal testing were also normal. Based on the symptoms and laboratory results, appendicitis was highly suspected, but it was important to distinguish it from other abdominal lesions like cholecystitis and intestinal ulcers. Therefore, computed tomography and color Doppler ultrasonography were performed, revealing thickening of the appendix without perforation or the obvious presence of a fecalith (**Figures 1A, B**). Considering the comprehensive examination findings and the 2020 WSES guidelines on acute appendicitis (Di Saverio et al., 2020), a preliminary diagnosis of acute appendicitis was established. Subsequently, an empirical anti-infective treatment with intravenous cefuroxime (1.5 g, three times a day) was started, aiming to prevent complications such as perforation or infection leading to peritonitis. The anti-infective treatment yielded improvement, with the patient’s abdominal pain being relieved the next day, but not completely. Consequently, upon the patient’s request and surgical indications, an appendectomy was performed via single-port laparoscopic surgery. The appendix was observed to be swollen and purulent, and a subsequent pathological examination confirmed the diagnosis of acute suppurative appendicitis and suggested the possibility of Gram-negative bacilli infection (**Figures 1C, D**). The patient continued to receive cefuroxime treatment and achieved complete recovery on the fifth day after admission (the abdominal pain symptoms, white blood cell count, and C-reactive protein were recovered normally), and then released from the hospital.

**Figure 2:**
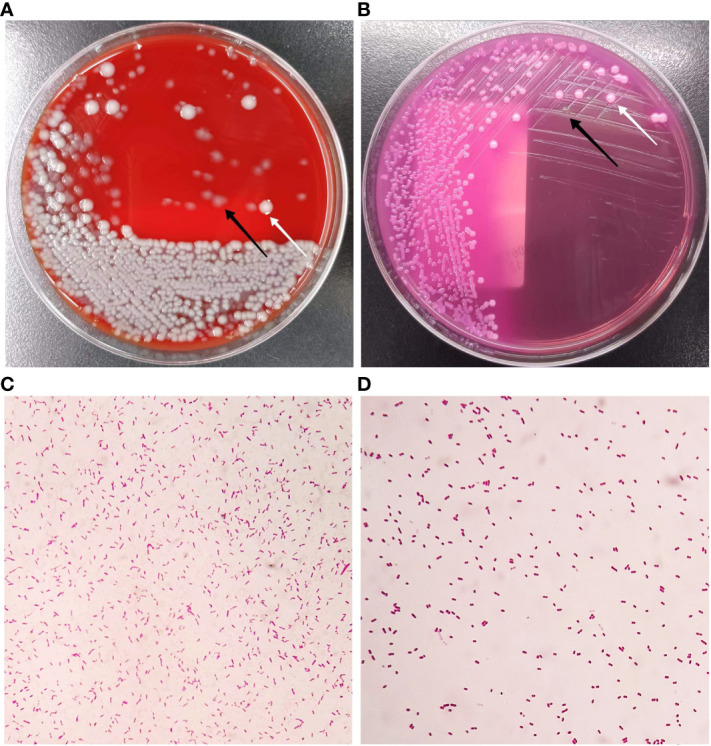
Colony characteristics of isolations from appendiceal purulent fluid. **(A)** Growth on Columbia blood agar after incubation at 35°C for 24 h. **(B)** Growth on MacConkey agar after incubation at 35°C for 24 h. **(C)** The Gram-stained morphology of *P. citronellolis* (2000X). **(D)** The Gram-stained morphology of *E. coli* (2000X). The white arrow indicates colonies of *E. coli*, and the black arrow indicates colonies of *P. citronellolis*.

The bacterial culture of the appendiceal purulent fluid was performed. Employing the loop with a 10 μL volume, we inoculated this sample onto Columbia blood agar and MacConkey agar media and incubated them at 35°C under aerobic and anaerobic conditions. After 24 hours of culture, we observed the growth of two distinct types of Gram-negative bacterial colonies on both Columbia blood agar and MacConkey agar media (**Figure 2**). The total bacterial counts exceeded 1×10^^6^ CFU/mL (≥+++) using a semi-quantitative approach (He, 2006; Klug et al., 2011) (**Figures 2A, B**). Next, we conducted identification of these isolates using matrix-assisted laser desorption/ionization time-of-flight mass spectrometry (MALDI-TOF MS) analysis, employing the Microflex LT instrument (Bruker Daltonics). In this analysis, we identified one of the bacteria as *E. coli*, scoring at 2.24 (>2.0 at the species level), and the other as *P. citronellolis*, scoring at 2.37. In our laboratory, the *P. citronellolis* isolate was misidentified as *Pseudomonas fluorescens* using the VITEK-2 system (BioMérieux) with an identification accuracy of 90%. Given the absence of biochemical phenotype data for the uncommon *P. citronellolis* in the VITEK-2 system database, we proceeded with 16S rRNA gene sequencing analysis to further validate its identification. This strain was submitted to GENEWIZ (Azenta Life Sciences), Inc., Suzhou, China, for identification. Sequencing procedures strictly adhered to the laboratory’s standard operating protocols, along with the primer information listed in **Supplementary Table 1**. In the GenBank database, the sequence of the *P. citronellolis* strains exhibited the highest similarity to our identified strain, with a remarkable 99.87% homology. The sequence data have been deposited in the GenBank database under accession number OR458572 (**Supplementary Sequence File**). Subsequently, we employed the VITEK-2 system to perform antibiotic susceptibility testing on these bacteria. The inhibitory concentration breakpoints were interpreted according to the current version (33rd Edition) of the Clinical Laboratory Standards Institute document M100 for sections of “Enterobacterales” and “Other Non-Enterobacterales” (https://clsi.org/[Accessed August 18, 2023]). The results revealed that *P. citronellolis* strain displayed sensitivity to piperacillin, piperacillin-tazobactam, second- to fourth-generation cephalosporins, meropenem, imipenem, levofloxacin, ciprofloxacin, gentamicin, amikacin, and tigecycline, and resistance to trimethoprim-sulfamethoxazole. The strain of *E. coli* demonstrated a high sensitivity to all the aforementioned antibiotics.

**Figure 3:**
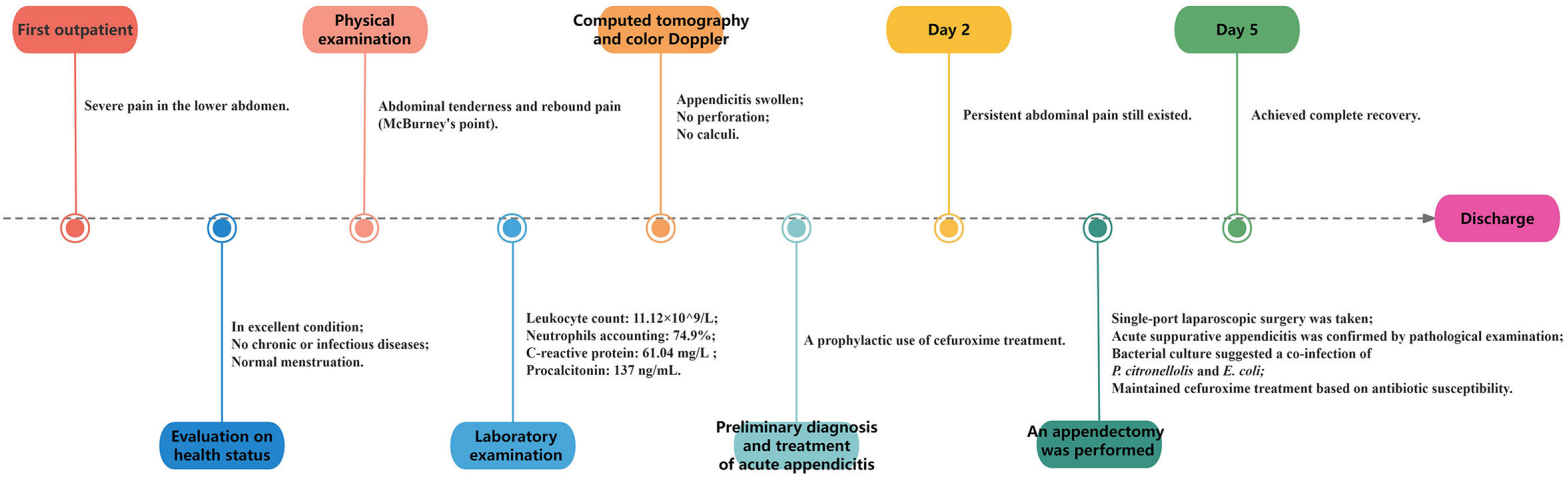
A timeline with relevant data from the episode of care.

A timeline with relevant data from the episode of care is shown in **Figure 3**.

## 3 Discussion and conclusions

*P. citronellolis* was initially found by Seubert in 1960 (Seubert, 1960). It is reported to primarily inhabit soil environments and display potential as a biocatalyst for terpene compounds (Seubert, 1960; Molina et al., 2013). However, its pathogenicity remains somewhat ambiguous. Clinical instances of *P. citronellolis* isolation are infrequent, with only two cases reported in recent years. In 2019, Williams. G first reported a case involving *P. citronellolis*-induced urinary tract infection and bacteremia. This case pertained to a 71-year-old male with a history of various conditions, including renal calculi, horseshoe kidney, diabetes, and elevated levels of prostate-specific antigen. Following a transrectal ultrasound-guided prostate biopsy, the patient developed a urinary tract infection accompanied by *P. citronellolis*-related bacteremia. Initially, this strain of *P. citronellolis* was misidentified as *Pseudomonas fluorescens*/*putida* through a phenotypic method using the MicroScan AutoSCAN system (with an identification rate of up to 95%). It was also incorrectly identified as *Pseudomonas fluorescens* using the Vitek 2 system. The correct identification was finally achieved through 16S rRNA gene sequencing and MALDI-TOF MS. This strain was sensitive to piperacillin-tazobactam, ceftriaxone, ceftazidimine, tobramycin, gentamicin, amikacin and tetracycline, but resistant to aztreonam, ciprofloxacin and trimethoprim-sulfamethoxazole (Williams, 2019). In 2023, Hwang CS reported another *P. citronellolis* infection case. An 80-year-old male had coronary artery disease, persistent atrial fibrillation, was undergoing warfarin therapy, and had comorbidities including congestive heart failure, type 2 diabetes, and a history of prostate cancer in remission. Additionally, he was diagnosed with myeloproliferative disorder and was undergoing treatment with the JAK inhibitor ruxolitinib. After experiencing the symptoms of gastroenteritis for several days, *P. citronellolis* and *Bacteroides thetaiotaomicron* were isolated from his blood. Due to compromised immune function, the patient might have experienced *P. citronellolis* and *Bacteroides thetaiotaomicron* translocation from the intestinal tract into the bloodstream following *Campylobacter* gastroenteritis (confirmed by Stool-PCR test), resulting in *P. citronellolis* and *Bacteroides thetaiotaomicron* bacteremia. Notably, multiplex PCR testing using the GenMark ePlex (Roche Diagnostics) initially couldn’t detect *P. citronellolis* but 16S rRNA gene sequencing and MALDI-TOF MS proved successful in identification. Moreover, *P. citronellolis* was not detected and verified in his intestinal tract. The isolate of *P. citronellolis* was susceptible to third- and fourth-generation cephalosporins, fluoroquinolones, aminoglycosides, carbapenems, piperacillin-tazobactam, and trimethoprim-sulfamethoxazole (Hwang et al., 2023).

In this report, we present a case of acute suppurative appendicitis caused by both *E. coli* and *P. citronellolis*. Notably, it is the first documented instance of *P. citronellolis*-associated appendicitis. Our patient was neither elderly nor immunocompromised. In this case, we deem that the isolated *P. citronellolis* was originated from the human body rather than environmental contamination. On the one hand, strict aseptic operation and wound disinfection, along with the employment of single-port laparoscopic minimally invasive surgery, effectively minimized exposure to the external environment. On the other hand, the bacterial count [exceeding 1×10^^6^ CFU/mL (≥+++)] exhibited distinct colonization and infection features. Given its anatomical structure, the appendix is a cul-de-sac connected to the cecum, acquiring bacteria mainly from intestinal contents including gut bacteria, foreign food, and feces. Therefore, it is necessary to consider *P. citronellolis* as a potential bacterium of intestinal content in patients. However, we highlight that *P. citronellolis* was unlikely to originate from a direct contamination of intestinal contents. Firstly, the absence of obvious fecalith or perforation (confirmed by imaging and subsequent pathological examination) led to the diagnosis of acute suppurative appendicitis, instead of acute obstructive or perforated appendicitis. Secondly, adhering to standard appendectomy procedures, the connection between the appendix base and the cecum was pre-ligated using an endoloop (Obrist et al., 2019), which effectively prevented the possibility of secondary infection and contamination due to intestinal content leakage.

It is worth noting that certain commercial biochemical/molecular identification systems, such as VITEK-2, MicroScan AutoSCAN, and GenMark ePlex do not accurately distinguish *P. citronellolis* from *Pseudomonas putida* or *Pseudomonas fluorescens* due to limited data regarding this bacterium (Williams, 2019; Hwang et al., 2023). This limitation was also evident in our study. However, our research demonstrated the utility of MALDI-TOF MS as a rapid and reliable tool for identifying rare environmental bacteria or clinical microorganisms, as previously reported (Tsuchida et al., 2020; Robert et al., 2021; Ashfaq et al., 2022). Current evidence suggests that this method allows for the accurate differentiation of *P. citronellolis* from other *Pseudomonas* species. The results from this approach were consistent with traditional morphological examinations and conclusive genetic sequencing, with identification scores consistently exceeding 2.0 at the species level. Importantly, in two additional studies, MALDI-TOF MS also successfully and accurately identified this strain at both genus and species levels (with scores >2.0) (Williams, 2019; Hwang et al., 2023). Thus, it is possible that the prevalence of *P. citronellolis* infections may have been underestimated in the past, particularly in scenarios where MALDI-TOF MS was not widely employed.

In conclusion, our study presents the first report of acute suppurative appendicitis related to bacterial co-infection with *P. citronellolis* and *E. coli*, occurring within a normal human host. In this case, *P. citronellolis* was first found to be associated with appendicitis, with a high bacterial load similar to the common *E. coli*. Considering the previous cases of infection and its high bacterial load, it’s crucial to recognize the potential pathogenicity of *P. citronellolis* for healthcare providers involved in appendicitis diagnosis and treatment. This finding underscores the bacterium’s capability to colonize the intestines of healthy individuals and potentially cause appendiceal infections. Furthermore, it also suggests the potential for infections to arise in other parts of the human intestine, supported by evidence that the bacterium was once isolated from the human rectum (source: https://www.ccug.se/[ Accessed August 18, 2023]). Indeed, the previous two cases also involved the intestine (transrectal ultrasound and gastroenteritis), although there was no direct evidence of isolation of *P. citronella* from the intestine (Williams, 2019; Hwang et al., 2023). Furthermore, it is important to acknowledge that our current comprehension of *P. citronellolis* remains limited. Given the scarcity of available reports, further research is needed to delve deeper into its pathogenicity, virulence, clinical infection, and environmental distribution. In this regard, the application of MALDI-TOF MS offers a novel perspective, providing an effective and accurate means for identifying infections caused by this bacterium in the clinic. This discovery not only broadens our understanding of the spectrum of infections associated with *P. citronellolis*, but also emphasizes the urgency of further research and attention in this area.

## Data availability statement

The datasets presented in this study can be found in online repositories. The names of the repository/repositories and accession number(s) can be found in the article/**Supplementary Material**.

## Ethics statement

The studies involving humans were approved and exempted by Ethics Committee of Zhongshan Bo’ai Hospital. The studies were conducted in accordance with the local legislation and institutional requirements. Written informed consent was obtained from the participant/patient(s) for the publication of this case report.

## Author contributions

YuL: Conceptualization, Formal analysis, Investigation, Methodology, Visualization, Writing – original draft. YaL: Formal analysis, Investigation, Visualization, Writing – review & editing. CL: Formal analysis, Investigation, Visualization, Writing – review & editing.
