# Supplementary material for: A case report of Pseudomonas citronellolis and Escherichia coli isolated from acute suppurative appendicitis: reveals the potential intestinal colonization and pathogenicity of Pseudomonas citronellolis
Source: Front Cell Infect Microbiol. 2024 Feb 16;14:1280188. doi: 10.3389/fcimb.2024.1280188 (PMC10904597; doi:10.3389/fcimb.2024.1280188)
Supplement: Supplementary file 1 [file DataSheet_1.docx]

**Supplementary Table 1** Information of 16S rRNA primers.

| **Variable region** | **Primer sequence** | **Annealing temperature** | **Length** |
| --- | --- | --- | --- |
| V3-V4 | Forward-5’-AGAGTTTGATCCTGGCTCA-3’ | 55℃ | 1500bp |
|  | Reverse-5’-GGTTACCTTGTTACGACTT-3’ |  |  |

**Supplementary Sequence File**

>*Pseudomonas citronellolis* strain 16S ribosomal RNA gene, partial sequence

AGAGTTTGATCCTGGCTCAGATTGAACGCTGGCGGCAGGCCTAACACATGCAAGTCGAGCGGATGAAGGGAGCTTGCTCCCTGATTCAGCGGCGGACGGGTGAGTAATGCCTAGGAATCTGCCTGGTAGTGGGGGACAACGTTCCGAAAGGAGCGCTAATACCGCATACGTCCTACGGGAGAAAGTGGGGGATCTTCGGACCTCACGCTATCAGATGAGCCTAGGTCGGATTAGCTAGTAGGTGGGGTAATGGCTCACCTAGGCGACGATCCGTAACTGGTCTGAGAGGATGATCAGTCACACTGGAACTGAGACACGGTCCAGACTCCTACGGGAGGCAGCAGTGGGGAATATTGGACAATGGGCGAAAGCCTGATCCAGCCATGCCGCGTGTGTGAAGAAGGTCTTCGGATTGTAAAGCACTTTAAGTTGGGAGGAAGGGCAGTAAGTTAATACCTTGCTGTTTTGACGTTACCAACAGAATAAGCACCGGCTAACTTCGTGCCAGCAGCCGCGGTAATACGAAGGGTGCAAGCGTTAATCGGAATTACTGGGCGTAAAGCGCGCGTAGGTGGTTTGGTAAGATGGATGTGAAATCCCCGGGCTCAACCTGGGAACTGCATCCATAACTGCCTGACTAGAGTACGGTAGAGGGTGGTGGAATTTCCTGTGTAGCGGTGAAATGCGTAGATATAGGAAGGAACACCAGTGGCGAAGGCGACCACCTGGACTGATACTGACACTGAGGTGCGAAAGCGTGGGGAGCAAACAGGATTAGATACCCTGGTAGTCCACGCCGTAAACGATGTCGACTAGCCGTTGGGATCCTTGAGATCTTAGTGGCGCAGCTAACGCGATAAGTCGACCGCCTGGGGAGTACGGCCGCAAGGTTAAAACTCAAATGAATTGACGGGGGCCCGCACAAGCGGTGGAGCATGTGGTTTAATTCGAAGCAACGCGAAGAACCTTACCTGGCCTTGACATGTCCGGAATCCTGCAGAGATGCGGGAGTGCCTTCGGGAATCGGAACACAGGTGCTGCATGGCTGTCGTCAGCTCGTGTCGTGAGATGTTGGGTTAAGTCCCGTAACGAGCGCAACCCTTGTCCTTAGTTACCAGCACGTTATGGTGGGCACTCTAAGGAGACTGCCGGTGACAAACCGGAGGAAGGTGGGGATGACGTCAAGTCATCATGGCCCTTACGGCCAGGGCTACACACGTGCTACAATGGTCGGTACAGAGGGTTGCCAAGCCGCGAGGTGGAGCTAATCCCAGAAAACCGATCGTAGTCCGGATCGCAGTCTGCAACTCGACTGCGTGAAGTCGGAATCGCTAGTAATCGTGAATCAGAATGTCACGGTGAATACGTTCCCGGGCCTTGTACACACCGCCCGTCACACCATGGGAGTGGGTTGCTCCAGAAGTAGCTAGTCTAACCGCAAGGGGGACGGTTACCACGGAGTGATTCATGACTGGGGTGAAGTCGTAACAAGGTAACC
